# Electronic reporting of rare endocrine conditions within a clinical network: results from the EuRRECa project

**DOI:** 10.1530/EC-23-0434

**Published:** 2023-11-20

**Authors:** S R Ali, J Bryce, A L Priego-Zurita, M Cherenko, C Smythe, T M de Rooij, M Cools, T Danne, H Katugampola, O M Dekkers, O Hiort, A Linglart, I Netchine, A Nordenstrom, P Attila, L Persani, N Reisch, A Smyth, Z Sumnik, D Taruscio, W E Visser, A M Pereira, N M Appelman-Dijkstra, S F Ahmed

**Affiliations:** 1 Developmental Endocrinology Research Group, School of Medicine, Dentistry & Nursing, University of Glasgow, Glasgow, UK; 2 Office for Rare Conditions, Royal Hospital for Children & Queen Elizabeth University Hospital, Glasgow, UK; 3 Department of Medicine, Division of Endocrinology, Leiden University Medical Center, Leiden, the Netherlands; 4 Department of Internal Medicine and Paediatrics, Ghent University, Belgium; 5 Department of Paediatric Endocrinology, Ghent University Hospital, Ghent, Belgium; 6 Diabetes Center AUF DER BULT, Hannover, Germany; 7 UCL GOS Institute of Child Health, London; 8 Department of Medicine & Clinical Epidemiology, Leiden University Medical Centre, Leiden, the Netherlands; 9 Division of Paediatric Endocrinology and Diabetes, Department of Paediatrics and Adolescent Medicine, University of Lübeck, Lübeck, Germany; 10 AP-HP, Université Paris Saclay, INSERM, Bicêtre Paris Saclay Hospital, le Kremlin Bicêtre, France; 11 Sorbonne Université, Inserm, Centre de recherche Sainte Antoine, APHP, Hôpital des Enfants Armand Trousseau, Paris, France; 12 Pediatric Endocrinology, Karolinska Institutet, Karolinska University Hospital, Stockholm, Sweden; 13 Clinical Genetics and Endocrinology Laboratory, Department of Laboratory Medicine, Semmelweis University, Budapest, Hungary; 14 Department of Endocrine and Metabolic Diseases, Istituto Auxologico Italiano IRCCS, Milan, Italy; 15 Department of Medical Biotechnology and Translational Medicine, University of Milan, Milan, Italy; 16 Endokrinologie, Medizinische Klinik Innenstadt und Poliklinik IV, Klinikum der Universität München, Munich, Germany; 17 Department of Pediatrics, Motol University Hospital and 2nd Faculty of Medicine, Charles University, Prague, Czech Republic; 18 National Centre for Rare Diseases, Istituto Superiore di Sanità, Rome, Italy; 19 Erasmus Medical Centre, Department of Internal Medicine, Academic Centre for Thyroid Diseases, Rotterdam, the Netherlands; 20 Department of Endocrinology and Metabolism, Amsterdam University Medical Centres, University of Amsterdam, Amsterdam, the Netherlands; 21 Amsterdam Gastroenterology Endocrinology and Metabolism, Amsterdam, the Netherlands

**Keywords:** registries, databases, rare conditions, rare diseases, European Reference Networks, endo-ERN

## Abstract

**Objective:**

The European Registries for Rare Endocrine Conditions (EuRRECa, eurreb.eu) includes an e-reporting registry (e-REC) used to perform surveillance of conditions within the European Reference Network (ERN) for rare endocrine conditions (Endo-ERN). The aim of this study was to report the experience of e-REC over the 3.5 years since its launch in 2018.

**Methods:**

Electronic reporting capturing new encounters of Endo-ERN conditions was performed monthly through a bespoke platform by clinicians registered to participate in e-REC from July 2018 to December 2021.

**Results:**

The number of centres reporting on e-REC increased to a total of 61 centres from 22 countries. A median of 29 (range 11, 45) paediatric and 32 (14, 51) adult centres had reported cases monthly. A total of 9715 and 4243 new cases were reported in adults (age ≥18 years) and children, respectively. In children, sex development conditions comprised 40% of all reported conditions and transgender cases were most frequently reported, comprising 58% of sex development conditions. The median number of sex development cases reported per centre per month was 0.6 (0, 38). Amongst adults, pituitary conditions comprised 44% of reported conditions and pituitary adenomas (69% of cases) were most commonly reported. The median number of pituitary cases reported per centre per month was 4 (0.4, 33).

**Conclusions:**

e-REC has gained increasing acceptability over the last 3.5 years for capturing brief information on new encounters of rare conditions and shows wide variations in the rate of presentation of these conditions to centres within a reference network.

**Significance statement**

Endocrinology includes a very wide range of rare conditions and their occurrence is often difficult to measure. By using an electronic platform that allowed monthly reporting of new clinical encounters of several rare endocrine conditions within a defined network that consisted of several reference centres in Europe, the EuRRECa project shows that a programme of e-surveillance is feasible and acceptable. The data that have been collected by the e-reporting of rare endocrine conditions (e-REC) can allow the continuous monitoring of rare conditions and may be used for clinical benchmarking, designing new studies or recruiting to clinical trials.

## Introduction

The field of clinical endocrinology covers a very wide range of rare conditions. However, clear information on the incidence and prevalence of these conditions is lacking. By pooling standardised information from several sources, registries have the potential to facilitate surveillance, audit and research. Generally, rare disease registries tend to focus more on the collection of detailed natural history data than support epidemiological research. On the other hand, linked datasets and population registries may not cover rare endocrine conditions at a sufficiently granular level to provide data on occurrence of specific diagnoses that can be compared at an international level and that are relevant to stakeholders such as patients, health-care professionals, health-care administrators and researchers (1).

Many clinical and scientific networks for rare conditions operate an electronic reporting system to perform surveillance as well as to understand the incidence and prevalence of conditions (2, 3, 4, 5). The European Registries for Rare Endocrine Conditions (EuRRECa) project was launched in 2018 (http://eurreb.eu) and aimed at providing its users with a wide range of registry solutions that would maximise the opportunity for patients, health-care professionals and researchers to participate in registries for rare endocrine conditions. Amongst these solutions, EuRRECa developed an electronic reporting system called e-reporting of rare endocrine conditions (e-REC, https://eurreb.eu/registries/e-rec/) which is a simple registry that can facilitate voluntary electronic surveillance of any activity within a network of centres such as the European Reference Network for rare endocrine conditions (Endo-ERN). These networks have a mission of objectively mapping conditions and related activity, aiming at better understanding the occurrence of the rare conditions covered within the network. Given that reference networks such as Endo-ERN have several centres, with several clinical users at each centre who may encounter a wide range of rare endocrine conditions, some that are more easily diagnosed than others, the e-REC platform was designed to capture with greatest ease as many conditions as possible. The current study reports the experience of e-REC over the three and a half years since its launch in 2018.

## Methods

### The e-REC platform

All centre leads of Endo-ERN were invited to register and use the e-REC platform. In addition, information about the platform was disseminated through allied professional societies including the European Society of Endocrinology (ESE) and the European Society for Paediatric Endocrinology (ESPE), with the platform open to all centres that look after people with such conditions. Between July 2018 and September 2019, data were collected and managed using the REDCap (Research Electronic Data Capture) tool hosted at the University of Glasgow (6). From October 2019, registered users were able to create a reporting set-up via a new bespoke web-based platform, also hosted at the University of Glasgow, in which the reporters could specify the rare endocrine conditions and the patient age group (patients under 18 years old and/or patients over 18 years old) that they would like to report on. Upon reporting a case, a unique ID was generated instantaneously for each case and provided electronically to users to be stored locally at the reporting centre. Multiple reporters could be selected within each reporting centre; however, a reporter from that centre was not able to sign up to report on the same condition within the same age group over the same reporting period as another reporter at the centre.

From the last quarter of 2019, users were able to specify whether a clinical diagnosis was suspected or confirmed and the platform also enabled updating of previously reported suspected cases to either a confirmed or excluded diagnosis. Reporters were invited to report any new case of any of the conditions included within Endo-ERN on a monthly basis and ‘the reporting month’ remained open for a period of three months. The reported data were stored on a secure server in the University of Glasgow and could be downloaded from the e-REC platform in MS Excel .CSV file format with details of the reporter, centre and information on the reported number of cases of each rare endocrine condition included within Endo-ERN. No personally identifiable information was collected for the reported cases and the process did not require informed patient consent. The project complied with EU GDPR (http://eur-lex.europa.eu/eli/reg/2016/679/oj) and was approved by the Information Governance authorities at the NHS Greater Glasgow & Clyde Health Board and the National Research Ethics Service in the UK. The reporting pattern of the centres that were in the top quintile (i.e. those that had reported in over 80% of the months since they joined the e-REC platform) was also studied separately to reduce the bias of intermittent reporting when studying overall annual reporting rate.

### Statistical analysis

Categorical data were analysed using descriptive statistics. Results are reported as frequencies and percentages and median (with ranges) values. Numerical data were collated and analysed using Minitab version 18 statistical software (Minitab LLC, State College, PA, USA).

## Results

### Reporting centres

**Figure 1:**
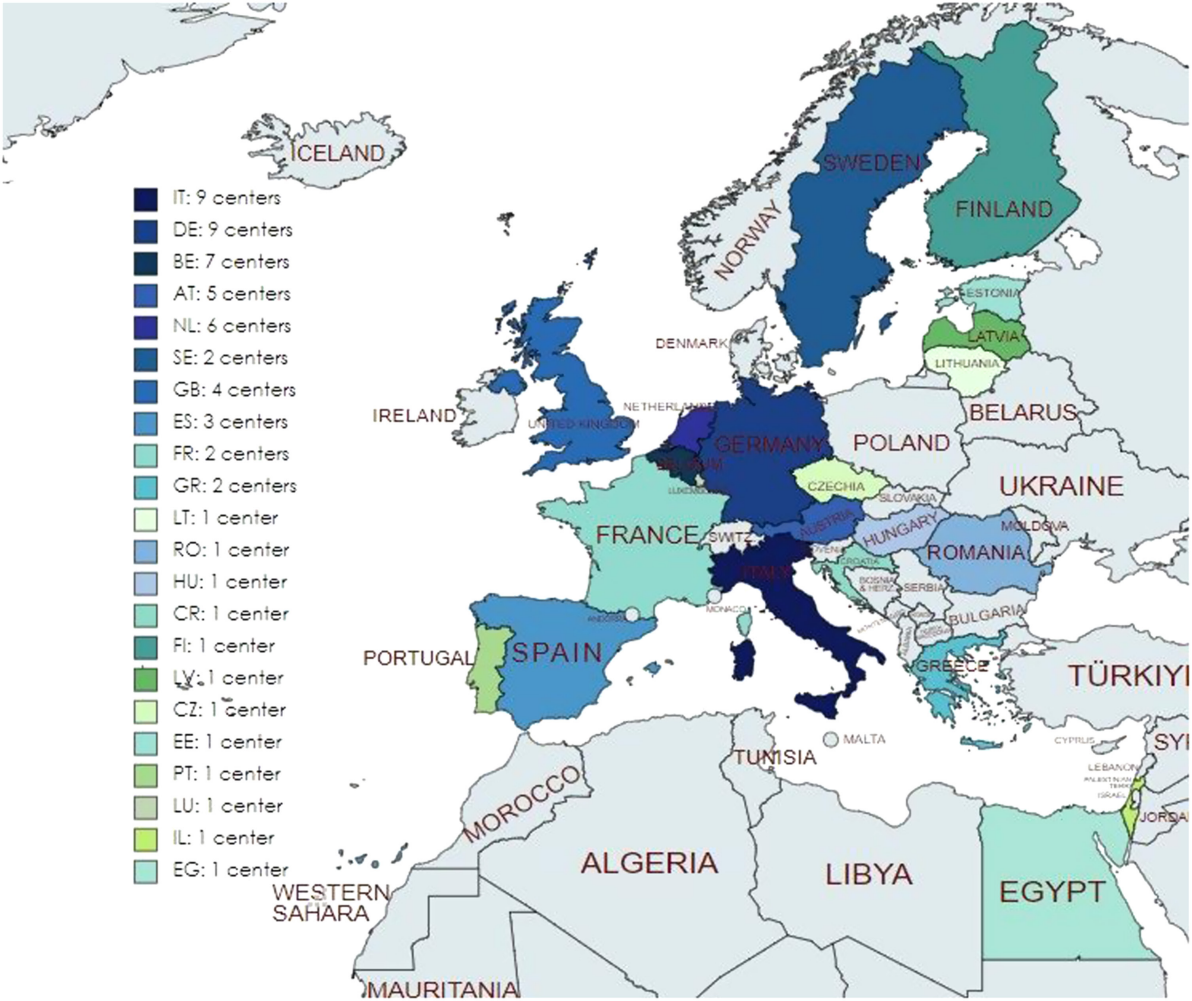
Centres reporting on the e-Reporting platform (e-REC) between July 2018 and December 2021. AT, Austria; BE, Belgium; CR, Croatia; CZ, Czechoslovakia; DE, Germany; EE, Estonia; EG, Egypt; ES, Spain; FI, Finland; FR, France; GB, UK; GR, Greece; HU, Hungary; IL, Israel; IT, Italy; LU, Luxembourg; LT, Lithuania; LV, Latvia; NL, Netherlands; PT, Portugal; RO, Romania; SE, Sweden.

**Figure 2:**
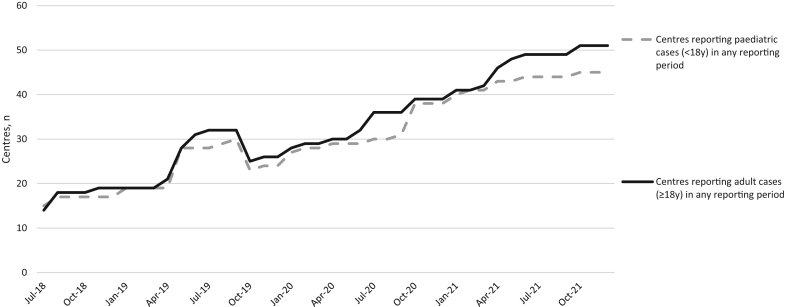
The change in the number of paediatric and adult centres reporting on the e-Reporting platform (e-REC) between July 2018 and December 2021.

Over the 3.5-year period, from July 2018 to December 2021, 61 centres from 22 countries had reported cases on e-REC (Fig. 1). Of these 61 centres, 44 (72%) were Endo-ERN reference centres, 3 (5%) were affiliated with another ERN (e.g. ERN-BOND) and 14 (23%) were not in any ERN. Of the 61 centres, 58 (95%) were from countries within the European Union. To date, 45 centres from 18 countries have reported paediatric cases (<18 years of age), 51 centres from 19 countries have reported adult cases (≥18 years of age) and 29 centres have reported on both children and adults. The cumulative number of centres (total centres that have submitted a case on e-REC) that had reported paediatric and adult cases increased over the 3.5-year period (Fig. 2). In December 2021, a median of 29 centres (range 11, 45) were reporting paediatric cases and 32 (14, 51) were reporting adult cases from 15 (10, 19) countries, on a monthly basis. Of the 61 centres, there were 33 centres (54%) who had reported in over 80% of the months since they joined the e-REC platform. Of these 33 centres, 23 were reporting adult cases and 25 were reporting paediatric cases (Table 1). Amongst the non-regular reporting centres, the median percentage of months these centres reported over the 3.5 years was 57% (5%, 79%).

**Table 1:** The number of cases reported from centres with 80% or more monthly submission rates from October 2019 to December 2021.

Endo-ERN MTG	Centres, *n*	Suspected cases reported, *n* (%)	Confirmed cases reported, *n* (%)	Total cases reported, *n*	Duration of reporting in months, median (range)	Cases reported per centre per month, median (range)	Estimated annual case reporting rate for a centre
**Children (<18 years)**							
Adrenal	17	42 (23%)	138 (77%)	180	21 (10, 27)	0.3 (0.1, 2)	3.6
Calcium and phosphate	18	30 (31%)	67 (69%)	97	19 (5, 27)	0.2 (0, 1.6)	2.4
Glucose and insulin	17	24 (15%)	141 (85%)	165	21 (1, 27)	0.1 (0, 3.1)	1.2
Genetic endocrine tumours	13	6 (9%)	60 (91%)	66	21 (2, 27)	0.1 (0, 1.3)	1.2
Growth and obesity	16	32 (12%)	225 (88%)	257	19 (5, 27)	0.4 (0, 5.6)	4.8
Pituitary	17	176 (51%)	168 (49%)	344	22 (4, 27)	0.4 (0, 8.7)	4.8
Sex development	20	133 (10%)	1230 (90%)	1363	22 (5, 27)	0.6 (0, 38)	7.2
Thyroid	17	89 (25%)	260 (75%)	349	22 (2, 27)	0.2 (0, 6.3)	2.4
**Adults (≥18 years)**							
Adrenal	17	230 (23%)	751 (77%)	981	24 (5, 27)	1.2 (0.3, 11.8)	14.4
Calcium and phosphate	16	12 (7%)	164 (93%)	176	21 (3, 27)	0.5 (0, 9.3)	6.0
Glucose and insulin	14	14 (36%)	25 (64%)	39	19 (5, 27)	0.02 (0, 0.8)	<1
Genetic endocrine tumours	16	61 (22%)	213 (77%)	274	25 (8, 27)	0.5 (0, 3.1)	6.0
Growth and obesity	9	1 (3%)	31 (97%)	32	21 (5, 27)	(0, 0, 1.5)	<1
Pituitary	17	295 (14%)	1789 (86%)	2084	24 (6, 27)	3.5 (0.4, 32.8)	42
Sex development	13	42 (5%)	772 (95%)	814	24 (8, 27)	1.8 (0, 8.6)	21.6
Thyroid	16	72 (7%)	917 (93%)	989	25 (5, 27)	2 (0, 9)	24

### Reported cases

**Figure 3:**
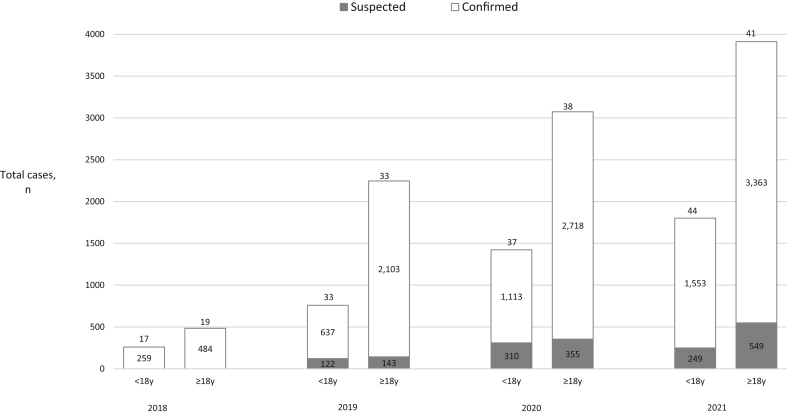
The change in the total number of cases reported on the e-Reporting platform (e-REC) between July 2018 and December 2021 in children and adults. The number at the top of each bar represents the number of centres that reported cases. The white and grey sections of each bar represent the number of confirmed and suspected cases, respectively.

**Figure 4:**
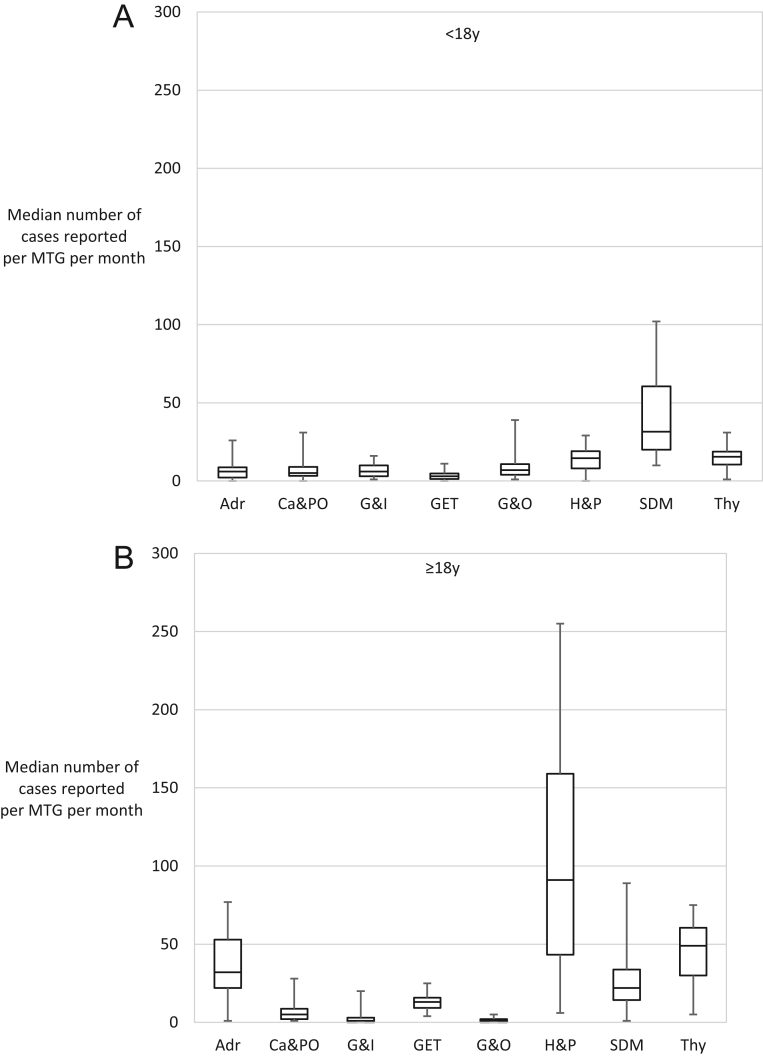
The total number of cases reported per main thematic group in children and adults between July 2018 and December 2021. The number at the top of each bar represents the number of centres that reported cases. Adr, adrenal; Ca&PO, calcium and phosphate; G&I, glucose and insulin; GET, genetic endocrine tumours; G&O, growth and obesity; H&P, hypothalamic and pituitary; SDM, sex development and maturation; Thy, thyroid.

**Figure 5:**
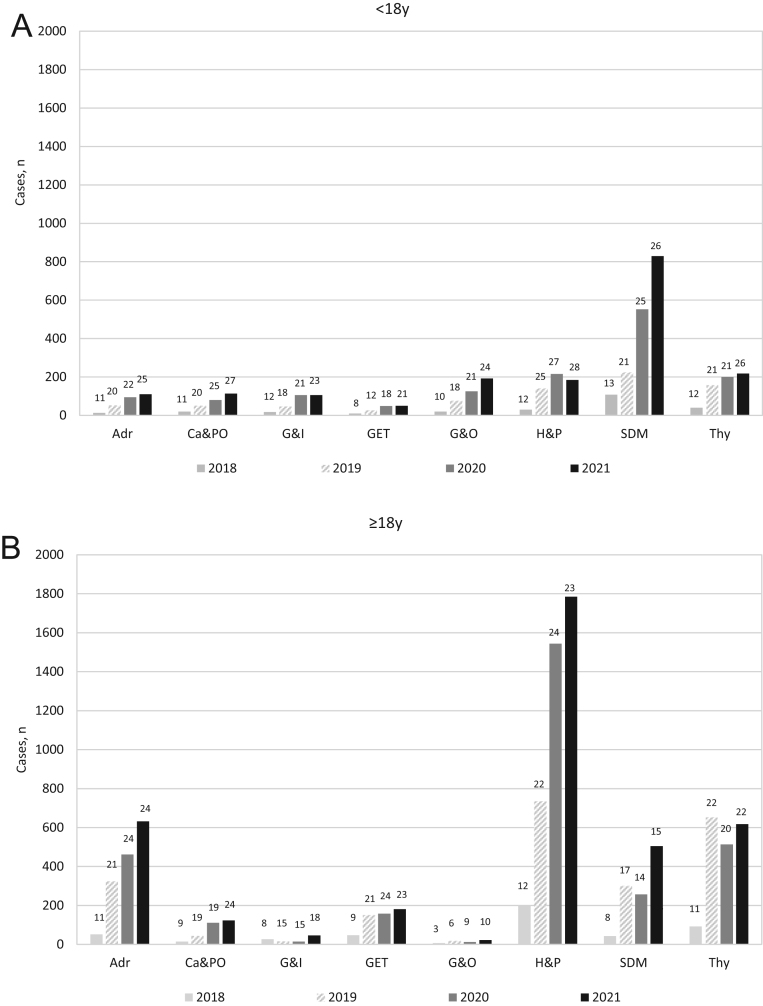
The median number of cases reported per main thematic group (MTG) per month between July 2018 and December 2021. Adr, adrenal; Ca&PO, calcium and phosphate; G&I, glucose and insulin; GET, genetic endocrine tumours; G&O, growth and obesity; H&P, hypothalamic and pituitary; SDM, sex development and maturation; Thy, thyroid.

Over the 3.5-year period, a total of 9715 and 4243 new cases were reported in adults and children (Table 2), respectively, with annual increases in the number of reported cases in both children and adults (Fig. 3). Overall, in adults and children, 8668 (89%) and 3562 (84%) cases were reported as confirmed and 1047 (11%) and 681 (16%) cases were reported as a suspected diagnosis, respectively (Fig. 3). In adults, an increase in the number of suspected diagnoses over time was also apparent. Moreover, there was a trend towards an increasing number of cases being reported on an annual basis for the majority of Endo-ERN condition groups (Fig. 4). The number of cases reported per condition group per centre also increased over the 3.5-year period (Fig. 5).

**Table 2:** The total number of cases reported within the eight Endo-ERN broad main thematic groups (MTG).

Endo-ERN MTGs (1-8)	Total number of cases reported, *n* (%)			
	<18 years		≥18 years	
**MTG 1. Adrenal**	268	6%	1470	15%
Congenital adrenal hyperplasia	181	68%	267	18%
Primary adrenal insufficiency	57	21%	287	20%
Cortisol-producing adenomas	12	4%	222	15%
Sporadic phaeochromocytoma-paraganglioma	12	4%	518	35%
Adrenocortical carcinomas	6	2%	164	11%
Familial hyperaldosteronism	0	0%	12	1%
**MTG 2. Calcium and phosphate**	262	6%	294	3%
X-linked hypophosphataemia	82	31%	31	11%
Pseudohypoparathyroidism	68	26%	10	3%
Hypoparathyroidism	43	16%	117	40%
Hyperparathyroidism including parathyroid cancer	16	6%	82	28%
Hypophosphataemic rickets	12	5%	6	2%
Hypocalcaemic vitamin D-dependent rickets	12	5%	5	2%
Familial hypocalciuric hypercalcaemia	9	3%	15	5%
PTH-independent hypercalcaemia	8	3%	12	4%
Hypocalcaemic vitamin D-resistant rickets	5	2%	2	1%
AD hypophosphataemic rickets	2	1%	3	1%
Familial hyperphosphataemic tumoural calcinosis	2	1%	4	1%
AR hypophosphataemic rickets	1	0%	0	0%
Hereditary hypophosphataemic rickets with hypercalciuria	1	0%	1	0%
Oncogenic osteomalacia	1	0%	6	2%
**MTG 3. Glucose and insulin**	274	6%	106	1%
Hyperinsulinism	176	64%	16	15%
Rare diabetes	89	32%	42	40%
Insulin resistance syndrome	9	3%	48	45%
**MTG 4. Genetic endocrine tumours**	134	3%	538	6%
MEN Type 1	47	35%	159	30%
MEN Type 2	41	31%	69	13%
Von Hippel–Lindau syndrome	24	18%	65	12%
Hereditary phaeochromocytoma-paraganglioma	14	10%	223	41%
Carney complex	8	6%	11	2%
Other neuroendocrine tumours	0	0%	11	2%
**MTG 5. Growth and obesity**	412	10%	61	1%
Rare genetic obesity	109	26%	2	3%
Prader–Willi syndrome and Prader–Willi-like syndrome	94	23%	49	80%
Noonan syndrome	87	21%	0	0%
Overgrowth syndrome	54	13%	1	2%
Silver–Russell syndrome	44	11%	6	10%
GH resistance syndromes	14	3%	0	0%
Beckwith–Wiedemann syndrome	10	2%	3	5%
ROHHAD syndrome	0	0%	0	0%
**MTG 6. Pituitary**	568	13%	4265	44%
Congenital hypopituitarism	324	57%	96	2%
Acquired hypopituitarism	179	32%	1199	28%
Pituitary adenoma	65	11%	2970	70%
**MTG 7. Sex development**	1713	40%	1106	11%
Transgender	988	58%	320	29%
XY DSD	294	12%	34	3%
Chromosomal DSD	259	11%	213	19%
XX DSD	90	4%	47	4%
Congenital normosmic hypogonadotrophic hypogonadism	52	2%	70	6%
Congenital anosmic hypogonadotrophic hypogonadism	30	1%	48	4%
**MTG 8. Thyroid**	612	14%	1875	19%
Congenital hypothyroidism	486	79%	18	1%
Nonmetastatic thyroid carcinoma	64	10%	1821	97%
Congenital hyperthyroidism	37	6%	0	0%
Thyroid hormone signalling disorders	25	4%	36	2%

In the 33 frequent reporting centres, total of 5389 cases were reported in adults and 2821 cases were reported in children. Of the 5389 cases in adults, 4662 (87%) were confirmed and 727 (14%) were suspected cases and in the 2821 cases in children, 2287 (81%) were confirmed and 532 (19%) were suspected (Table 1). Amongst the frequently reporting centres, the percentage of suspected cases varied from one condition group to another and amongst children, pituitary and calcium disorders comprised the greatest number of suspected cases, whilst in adults, disorders of glucose and insulin homeostasis comprised the highest percentage of suspected cases (Table 1).

### Adrenal disorders

Of the 4243 new cases in children, 268 (6%) had an adrenal disorder. Of these 268, 181 (68%) were cases of congenital adrenal hyperplasia and 57 (21%) were primary adrenal insufficiency (Table 2). Of the 9715 cases in adults, adrenal disorders were reported in 1470 (15%) with 518 cases (15%) of sporadic phaeochromocytoma-paraganglioma. The overall median (range) number of paediatric and adult cases reported by centres for adrenal conditions was 6 (0, 26) and 32 (1, 77), respectively (Fig. 5). In children, despite an initial increase over the first 1.5 years of reporting, the total number of adrenal cases reported remained constant over the latter 2-year period, whilst the number of adrenal cases reported in adults continued to increase (Fig. 4). The estimated annual case reporting rate amongst the 33 frequently reporting centres for children and adults was 3.6 per centre and 14.4 per centre, respectively (Table 1).

### Disorders of calcium and phosphate homeostasis

In children, of the 4243 new cases, 262 (5%) calcium and phosphate conditions were reported; 82 (31%) were specified as X-linked hypophosphataemia, 68 (26%) as pseudohypoparathyroidism and 43 (16%) as hypoparathyroidism; these conditions comprised more than 70% of reported cases in this condition group (Table 2). Of the 9715 cases in adults, 294 (3%) had calcium and phosphate conditions, with 117 (40%) cases of hypoparathyroidism and 82 (28%) cases of hyperparathyroidism. The overall median number of paediatric and adult cases reported by centres for this group of conditions was 5 (0, 31 and 1, 28), respectively and the total number of cases reported over the past 2 years in both groups remained constant. (Figs. 4 and 5) The estimated annual case reporting rate amongst the frequently reporting centres for children and adults was 2.4 per centre and 6.0 per centre, respectively (Table 1).

### Genetic disorders of glucose and insulin homeostasis

Of the 4243 new cases in children, 274 (5%) were glucose disorders with 176 (64%) cases of hyperinsulinism (HI), 89 (32%) of rare diabetes and 9 (3%) of insulin resistance syndrome (Table 2). These conditions were less frequently reported in adults and of 9715 cases, 16 cases (0.2%) of HI, 42 cases (0.4%) of rare diabetes and 48 cases (0.5%) of insulin resistance syndrome were specified. The overall median number of paediatric and adult cases reported by centres for glucose and insulin disorders was 6 (1, 14) and 1 (0, 20), respectively (Fig. 5). The estimated annual case reporting rate amongst the 33 frequently reporting centres for children and adults was 1.2 per centre and <1 per centre, respectively (Table 1).

### Genetic endocrine tumour syndromes

In children, of the 4243 cases, genetic endocrine tumour syndromes were reported in 134 (3%) of all cases and comprised the smallest number of reported conditions of all Endo-ERN condition groups (Table 2). Multiple endocrine neoplasias (MEN) accounted for more than 75% of reported cases, with 47 (35%) cases of MEN type 1 and 41 (31%) cases of MEN type 2 specified. In adults, of the 9715 cases, there were 538 (6%) cases of genetic tumour syndromes; hereditary phaeochromocytoma-paraganglioma and MEN type 1 were the most commonly reported conditions, comprising 223 (41%)) and 159 (30%) conditions, respectively. The overall median number of paediatric and adult cases reported by centres for genetic endocrine syndrome was 3 (0, 11) and 13 (4, 25), respectively (Fig. 5). The estimated annual case reporting rate amongst the 33 frequently reporting centres for children and adults was 1.2 per centre and 6.0 per centre, respectively (Table 1).

### Growth and genetic obesity syndromes

In children, of the 4243 cases, 412 (10%) growth and rare genetic obesity conditions were reported. Rare genetic obesity, Prader–Willi syndrome and Noonan syndrome, were reported in 109 (26%), 94 (23%) and 87 (21%) cases, respectively, comprising 70% of reported growth conditions (Table 2). In adults disorders of growth and obesity were the least commonly reported condition group and of the 9715 conditions reported in adults, only 61 (1%) cases of this group were reported, with 49 (80%) cases attributed to Prader–Willi syndrome. The overall median number of paediatric and adult cases reported by centres for growth conditions was 7 (1, 39) and 1 (0, 5), respectively (Fig. 5). The estimated annual case reporting rate amongst the 33 frequently reporting centres for children and adults was 4.8 per centre and <1 per centre, respectively (Table 1).

### Hypothalamic and pituitary disorders

In children, of the 4243 cases, 568 (13%) pituitary conditions were reported, with 324 (57%) cases of congenital hypopituitarism and 179 (32%) cases of acquired hypopituitarism reported (Table 2). In adults, conditions within the pituitary group were most commonly reported. Of the 9715 cases in adults, 4265 (44%) pituitary cases were reported. with an increasing number of cases reported over time (Fig. 4). The most common pituitary condition reported in adults was pituitary adenoma, with 2970 (70%) cases reported over a 3.5-year period. The median number of cases reported by centres for pituitary conditions in children was 15 (0, 29) and 91 (6, 255) in adults (Fig. 5). The estimated annual case reporting rate amongst the 33 frequently reporting centres for children and adults was 4.8 per centre and 42 per centre, respectively (Table 1).

### Sex development and maturation disorders

In children, conditions within the sex development group were most commonly reported, comprising 1713 (40%) cases of a total of 4243 reported cases (Table 2). Cases of transgender were reported in 988, comprising 58% of reported cases in this condition group. In adults, of the 9715 reported cases, 1106 (11%) of all reported cases were sex development disorders, with 694 (62%) cases of transgender. The median number of paediatric and adult cases reported by centres for conditions within the sex development were 32 (10, 102) and 22 (1, 89) (Fig. 5). A trend towards an increasing number of cases being reported over time was apparent in both age groups (Fig. 4). The estimated annual case reporting rate amongst the 33 frequently reporting centres for children and adults was 7.2 per centre and 21.6 per centre, respectively (Table 1).

### Thyroid disorders

In children, of the 4243 reported conditions, 612 cases of thyroid conditions were reported, comprising 14% of all reported cases in children (Table 2). Congenital hypothyroidism was the most commonly reported condition in this group with 486 (79%) cases reported. In adults, of the 9715 conditions reported, 1875 (19%) thyroid cases were reported. Non-metastatic carcinoma was the commonest condition and reported in 1821 (97%) adult cases. The median number of cases reported by paediatric and adults centres for thyroid conditions were 16 (1, 31) and 49 (5, 75), respectively, with the overall number of reported cases remaining constant over time (Fig. 4 and 5). The estimated annual case reporting rate amongst the 33 frequently reporting centres for children and adults was 2.4 per centre and 24 per centre, respectively (Table 1).

## Discussion

Since its launch in 2018, the e-REC platform, an electronic reporting platform within the European Registries for Rare Endocrine Conditions (EuRRECa) project has enabled monthly reporting of new clinical cases by 61 centres from 22 countries, clearly showing that the platform can function as a registry which can perform simple epidemiological surveillance. The results show a steady increase in the total number of centres actively reporting cases via e-REC over the past 3.5 years. Around 30% of the reporting centres were also outside the Endo-ERN network, demonstrating the wider scope of this platform for multicentre international collaboration within the endocrine community.

This initial phase in the use of e-REC has also seen an increase in the total number of cases of rare endocrine conditions reported, across most broad groups of conditions, in both adults and children. This may reflect widening participation, with a greater number of centres actively reporting cases over time and increasing awareness of the e-REC platform and Endo-ERN activities amongst the wider endocrine community. Despite comparable numbers of centres reporting paediatric and adult cases, a higher number of total cases were reported in adults overall (9715 vs 4243 cases). It is possible that a higher number of cases in adults may be due to a higher incidence and prevalence of these conditions in adults or due to some specific rare conditions which are commoner in adults. Previous studies examining the epidemiology of more common endocrine disorders in an industrialised nation estimated a prevalence of about 5% for some conditions including disorders of glucose homeostasis, calcium and phosphate conditions and thyroid disorders in adults, with a much lower incidence for other conditions including adrenocortical carcinoma, phaeochromocytoma, and pituitary adenomas (7). Although the epidemiology of more common conditions such as diabetes has been well-defined in large population-based studies (8), there is a lack of comprehensive data regarding population-based estimates of the prevalence of many rarer endocrine conditions. By examining the centres that reported regularly and frequently, we have also estimated the annual reporting rates of rare endocrine conditions presenting to centres within a network such as Endo-ERN. We believe that these figures can, therefore, be reliably used as a clinical benchmark for evaluating reporting rates amongst centres. These reporting rates should not be viewed as prevalence rates of these rare conditions as that would require a more population-focussed study; however, they do provide an estimate of the utilisation of health-care services. Given that the referral patterns of individual reference centres are very variable, using the reported rate for understanding prevalence will be challenging. Further studies that may influence differences in prevalence and presentation of these conditions to health care providers are vital due to implications for planning of health service delivery models and allocation of public health and research resources (9).

Our results show that some rare endocrine conditions were more commonly reported than others. Overall, in children, almost half of all reported cases were conditions affecting sex development, with transgender cases comprising 58% of reported cases; recent studies have also reported increasing rates of transgender cases amongst both age groups (10, 11). In adults, almost half of total reported cases were pituitary conditions with pituitary adenomas comprising 70% of these reported cases. These data are similar to those reported by Endo-ERN in 2018 in an internal exercise (12). However, challenges still exist in estimating the prevalence of pituitary adenomas and wide variation has been reported (13, 14).

The current bespoke e-REC platform enables reporting of confirmed and/or suspected cases which is particularly useful in cases where genetic or biochemical diagnostic confirmation is pending. Overall, in children and adults, a greater proportion of reported cases were defined as having a confirmed diagnosis rather than a suspected diagnosis. Interestingly, our results show that a greater proportion of cases were reported as suspected in children compared with adults, and this may reflect the groups of conditions that are more often reported in these two age groups. It is also possible that different centres have a different threshold for reporting the same condition as suspected or confirmed. Given that standardised diagnostic criteria and improvements in diagnostic ability should lead to a greater proportion of cases being reported as confirmed, rather than suspected, the prospective use of e-REC will allow networks such as Endo-ERN to monitor this metric of care.

Limitations that should be acknowledged include potential response bias amongst reporting centres, differences in the definition of a confirmed and suspected condition and the collection of a small core dataset with information only collected on details of the reporter, reporting centre and the number of cases of each rare endocrine condition reported. However, it was imperative to reduce respondent burden as reporters were expected to complete case returns on monthly basis. Although COVID-19-related lockdown could have hampered the reporting of rare endocrine conditions, our data show that this did not appear to be the case amongst participating centres. Going forward, it would also be helpful to compare the reported data to the actual hospital activity data to ascertain the level of reporting accuracy and this could be performed on an occasional basis as a quality assurance exercise. Another possible limitation includes the preponderance of participating centres from Europe, with 95% of centres from within the EU.

In addition to being a registry platform that minimises reporting burden, the platform can also be adapted to serve the needs of several networks that are interested in understanding the occurrence of multiple rare conditions or events. For instance, during the recent COVID-19 pandemic, the e-REC platform was used to collect essential data concerning specific groups of patients with rare endocrine conditions, who were also affected by COVID-19 (15). Thus, e-REC can be used for continued surveillance of conditions or for a fixed period. In summary, the e-REC platform is a simple online platform that can be used to capture information on new encounters of patients with rare endocrine conditions that present to participating centres.

## Declaration of interest

Faisal S Ahmed is a senior editor of *Endocrine Connections*. Faisal S Ahmed was not involved in the review or editorial process for this paper, on which he is listed as an author.

## Funding

S.F.A., J.B. and L.P. are supported by the European Union’s Health Programme (2014–2020) on the EuRRECa project ‘777215/EuRRECa’. S.F.A., N.M.A-D. and A.P.Z. are supported by the European Union’s Health Programme (2014–2020) on the EuRR-Bone project ‘946831/EuRR-Bone’. The EuRRECa and Endo-ERN projects are also grateful to the European Society of Endocrinology http://dx.doi.org/10.13039/100010382 and the European Society for Paediatric Endocrinology http://dx.doi.org/10.13039/100010381 for funding support. A.M.P. is supported by the European Reference Network on Rare Endocrine Conditions (Endo-ERN). Endo-ERN is funded by the European Union within the framework of the EU4H Programme, grant agreement number: 101084921.

## Data availability

The data that support the findings of this study are available from the corresponding author upon reasonable request.

## References

[1] Crafa A Calogero AE Cannarella R Mongioi LM Condorelli RA Greco EA Aversa A & La Vignera S. The burden of hormonal disorders: a worldwide overview with a particular look in Italy. Frontiers in Endocrinology 2021 12 694325. (10.3389/fendo.2021.694325) 34220719 PMC8242938

[2] Lynn RM Pebody R & Knowles R. Twenty years of active paediatric surveillance in the the UK and Republic of Ireland. Euro Surveillance 2006 11 E060720.4. (10.2807/esw.11.29.03005-en) 16966765

[3] Elliott EJ Nicoll A Lynn R Marchessault V Hirasing R & Ridley G. Rare disease surveillance: an international perspective. Paediatrics and Child Health 2001 6 251–260. 20084246 PMC2804555

[4] El-Fakhri N Williams C Cox K McDevitt H Galloway P McIntosh N & Ahmed SF. An electronic surveillance system for monitoring the hospital presentation of nutritional vitamin D deficiency in children in Scotland. Journal of Pediatric Endocrinology and Metabolism 2013 26 1053–1058. (10.1515/jpem-2013-0175) 23828491

[5] Rodie ME Ali SR Jayasena A Alenazi NR McMillan M Cox K Cassim SM Henderson S McGowan R Ahmed SF, *et al.* A nationwide study of the prevalence and initial management of atypical genitalia in the newborn in Scotland. Sexual Development 2022 16 11–18. (10.1159/000517327) 34352789

[6] Harris PA Taylor R Minor BL Elliott V Fernandez M O'Neal L McLeod L Delacqua G Delacqua F Kirby J, *et al.* The REDCap consortium: building an international community of software platform partners. Journal of Biomedical Informatics 2019 95 103208. (10.1016/j.jbi.2019.103208) 31078660 PMC7254481

[7] Golden SH Robinson KA Saldanha I Anton B & Ladenson PW. Clinical review: prevalence and incidence of endocrine and metabolic disorders in the United States: a comprehensive review. Journal of Clinical Endocrinology and Metabolism 2009 94 1853–1878. (10.1210/jc.2008-2291) 19494161 PMC5393375

[8] Ford ES Li C Zhao G Pearson WS & Mokdad AH. Prevalence of the metabolic syndrome among U.S. adolescents using the definition from the International Diabetes Federation. Diabetes Care 2008 31 587–589. (10.2337/dc07-1030) 18071007

[9] Golden SH Brown A Cauley JA Chin MH Gary-Webb TL Kim C Sosa JA Sumner AE & Anton B. Health disparities in endocrine disorders: biological, clinical, and nonclinical factors--an Endocrine Society scientific statement. Journal of Clinical Endocrinology and Metabolism 2012 97 E1579–E1639. (10.1210/jc.2012-2043) 22730516 PMC3431576

[10] Indremo M White R Frisell T Cnattingius S Skalkidou A Isaksson J & Papadopoulos FC. Validity of the gender dysphoria diagnosis and incidence trends in Sweden: a nationwide register study. Scientific Reports 2021 11 16168. (10.1038/s41598-021-95421-9) 34373498 PMC8352918

[11] de Graaf NM Carmichael P Steensma TD & Zucker KJ. Evidence for a change in the sex ratio of children referred for gender dysphoria: data from the gender identity development service in London (2000–2017). Journal of Sexual Medicine 2018 15 1381–1383. (10.1016/j.jsxm.2018.08.002) 30195563

[12] de Vries F Bruin M Cersosimo A van Beuzekom CN Ahmed SF Peeters RP Biermasz NR Hiort O & Pereira AM. An overview of clinical activities in Endo-ERN: the need for alignment of future network criteria. European Journal of Endocrinology 2020 183 141–148. (10.1530/EJE-20-0197) 32413847

[13] Melmed S. Pituitary-tumor endocrinopathies. New England Journal of Medicine 2020 382 937–950. (10.1056/NEJMra1810772) 32130815

[14] Molitch ME. Diagnosis and treatment of pituitary adenomas: a review. JAMA 2017 317 516–524. (10.1001/jama.2016.19699) 28170483

[15] Nowotny HF Bryce J Ali SR Giordano R Baronio F Chifu I Tschaidse L Cools M Van den Akker ELT Falhammar H, *et al.* Outcome of COVID-19 infections in patients with adrenal insufficiency and excess. Endocrine Connections 2023 12 e220416. (10.1530/EC-22-0416) PMC10083676 36715679

